# The First Case of Daratumumab-Induced Fulminant Hepatic Failure

**DOI:** 10.7759/cureus.46858

**Published:** 2023-10-11

**Authors:** Ali Tariq Alvi, Sachin George Mathew, Murali Shankar

**Affiliations:** 1 Internal Medicine, HCA Florida Westside Hospital, Plantation, USA; 2 Internal Medicine, HCA Florida Northwest Hospital, Margate, USA; 3 Gastroenterology, HCA Florida Westside Hospital, Plantation, USA

**Keywords:** acute fulminant liver failure, chemotherapy, thrombocytopenia, daratumumab, drug induced

## Abstract

Drug-induced liver failure is a relatively uncommon condition with a vast spectrum of clinical manifestations, and it is a leading cause of acute hepatic failure in the United States. We describe the first case of fulminant hepatic failure induced by chemotherapeutic drug daratumumab, a common FDA-approved agent. A 77-year-old male, with a history of multiple myeloma, was admitted for left lower extremity cellulitis, two weeks after receiving his first intravenous infusion of daratumumab. He developed fulminant hepatic failure in the hospital a few days later. Despite multiple doses of N-acetylcysteine, his liver function continued to decline, and he expired shortly after.

## Introduction

Daratumumab, which received U.S Food and Drug Administration (FDA) approval in 2015, is the first humanized monoclonal antibody targeted against cell surface receptor CD38 and is commonly used in the treatment of multiple myeloma [1]. It is generally well-tolerated as monotherapy and as combination therapy. The most common adverse effects include nausea, fatigue, back pain, anemia, cough, upper respiratory tract infection, thrombocytopenia, and neutropenia. Infusion-related reactions (IRRs) occur in approximately 48% of patients, usually during the first dose, and include cough, nasal congestion, rhinorrhea, throat irritation, and dyspnea [2]. Most of these IRRS are mild and can be managed with pre- and post-treatment with acetaminophen, antihistamine, and corticosteroids [3].

Drug-induced liver injury is a relatively uncommon clinical event with an estimated incidence between 2.3 and 13.9 cases per 100,000 people in Europe. However, it carries a significant risk of morbidity and mortality, making it a leading cause of acute hepatic damage in the United States [4]. Hepatic toxicity has been reported with many oncologic and non-oncologic medications in the literature. To date, hepatic toxicity associated with daratumumab has not been reported. We present the first case report of daratumumab-induced fulminant liver failure, leading to acute encephalopathy and eventually death of the patient.

## Case presentation

**Figure 1:**
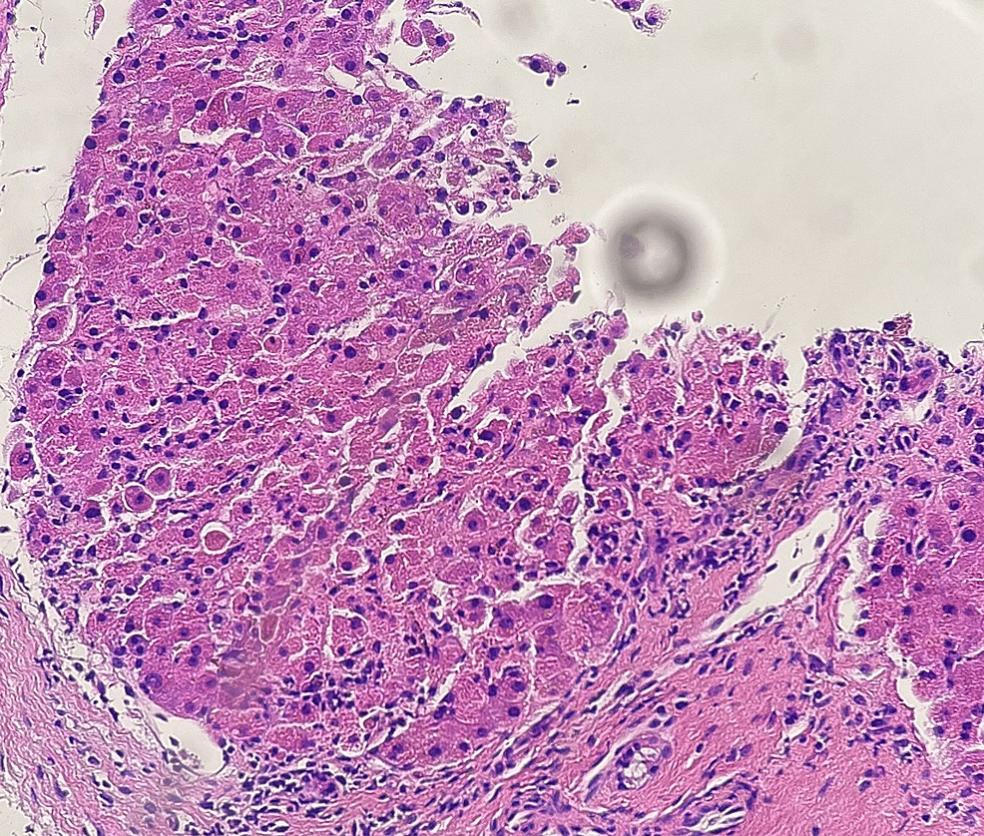
Histopathology of the liver showing acute hepatic injury with individual cell necrosis and mononuclear infiltrate.

We describe a 77-year-old male, with a past medical history of hypertension, hyperlipidemia, type two diabetes mellitus, multiple myeloma, end-stage renal disease on hemodialysis, and hepatitis C treated 10 years ago, who presented in the emergency department with left lower extremity pain with erythema. Clinical presentation was consistent with cellulitis, and the patient was started on vancomycin and cefepime. Blood cultures were unremarkable, but wound cultures grew *Serratia marcescens*. The patient had no prior history of liver failure, with normal hepatic function on prior laboratory tests. Laboratory tests in hospital revealed white blood cell (WBC) count of 8,200/ml, hemoglobin of 8.8 g/dl, platelet count of 124,000/mL, alanine aminotransferase (ALT) of 101 U/L, aspartate aminotransferase (AST) of 358 U/L, alkaline phosphatase (ALP) of 172 U/L, ferritin of 443 ng/ml, total bilirubin of 1.4 mg/dl, albumin of 3.2 g/dl, and negative antinuclear antibody (ANA). In the next few days, the liver function tests and platelet count continued to worsen. Acetaminophen levels were non-toxic, and the hepatitis panel only revealed prior hepatitis C infection. Ultrasound of the abdomen only showed non-specific gall bladder wall thickening. The coagulation profile on day seven revealed prothrombin time (PT) of 63.9 seconds, international normalized ratio (INR) of 5.1, activated partial thromboplastin time (aPTT) of 107 seconds, fibrinogen of 173 mg/dL, factor II of 37%, factor VII of 9%, factor VIII of 480%, factor IX of 31%, and factor X of 21.7%. A liver biopsy was performed, which revealed acute hepatic injury, with individual cell necrosis and periportal mononuclear infiltrate (Figure 1).

**Figure 2:**
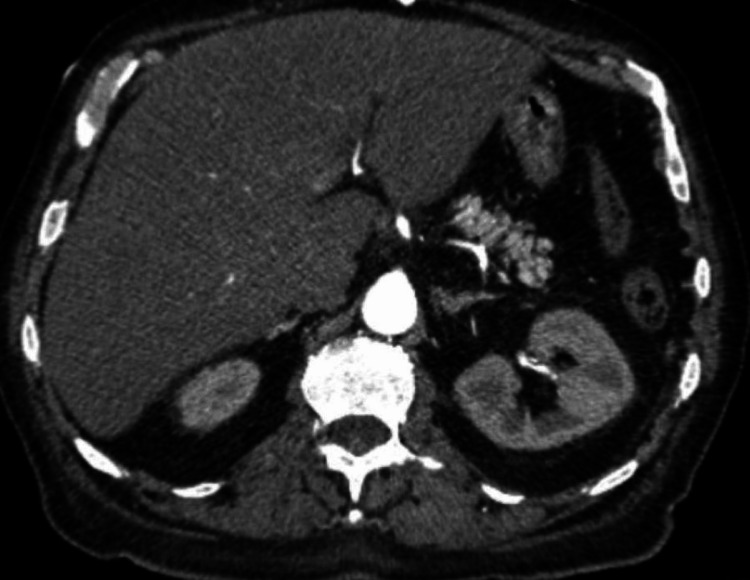
Computed tomography (CT) scan of the abdomen showing normal liver parenchyma and size.

Moreover, it was negative for steatohepatitis or cirrhosis. Herpes simplex virus (HSV) and cytomegalovirus (CMV) stains were also negative. Computed tomography (CT) scan of abdomen showed splenic atrophy, but normal liver and gall bladder size (Figure 2).

Therefore, it was assumed that acute hepatic failure was presumably related to recent medication use due to unremarkable other investigations. There was no recent change in home medications, which included metoprolol, pravastatin, sertraline, pantoprazole, calcitriol, cyanocobalamin, and insulin glargine. The patient was given three doses of intravenous 20% N-acetylcysteine, in an attempt to prevent hepatic damage. Despite this therapy, his liver function tests and thrombocytopenia continued to worsen (Table 1).

**Table 1:** Trend of laboratory test results during hospitalization.

	Day 5	Day 6	Day 7	Day 8	Reference Ranges
Complete blood count (CBC)					
Hemoglobin	9.3	9.8	10.1	11.1	13.7-17.5 g/dl
White blood cell (WBC)	10.3	10.6	14.1	16.8	4.0-10.5 x 10^3^/µL
Platelets	56	61	44	41	150-400 x 10^3^/µL
Comprehensive metabolic panel (CMP)					
Blood urea nitrogen (BUN)	32	16	19	12	6-22 mg/dl
Creatinine	5.3	3.5	4.99	3.1	0.43-1.13 mg/dl
Sodium	124	126	125	125	135-145 mmol/L
Potassium	4.4	3.7	3.3	3.3	3.5-5.2 mmol/L
Chloride	88	92	91	94	95-110 mmol/L
Bicarbonate	21	23	16	20	19-34 mmol/L
Aspartate aminotransferase (AST)	1,221	1,744	2,620	3,201	10-40 U/L
Alanine aminotransferase (ALT)	340	467	664	746	10-60 U/L
Alkaline phosphatase (ALP)	202	249	202	339	20-130 U/L
Total bilirubin	3.2	4.7	6.0	7.0	0.1-1.2 mg/dl
Albumin	3.1	2.9	2.6	2.4	3.2-5.0 g/dl

The patient initially developed encephalopathy and expired a few days later. On review of recent medication changes, it was found that the patient received the first intravenous daratumumab infusion two weeks ago for multiple myeloma.

## Discussion

Daratumumab is an immunoglobulin G1 kappa (IgG1k) human monoclonal antibody that is approved by the FDA for the management of multiple myeloma as monotherapy and in combination with bortezomib, lenalidomide, or pomalidomide for refractory or relapsed cases. It is also approved to be used in combination with medications including bortezomib, lenalidomide, dexamethasone, or only dexamethasone for newly diagnosed cases of multiple myeloma [1]. This antibody targets CD38, the cell surface antigen, and induces cell death through apoptosis, as well as antibody-mediated phagocytosis and cytotoxicity [5]. To our knowledge, this is the first reported case of daratumumab-induced acute hepatitis, leading to metabolic encephalopathy and soon after the patient's demise.

Acute liver failure, also referred to as fulminant hepatitis or acute hepatic necrosis, is characterized by acute liver injury, hepatic encephalopathy, and an international normalized ratio of >1.5, with an illness duration of >26 weeks [3]. It can further be classified into hyperacute (within hours) and acute or subacute (within days to weeks) depending on the time from the onset of symptoms to the onset of hepatic encephalopathy. There are numerous etiologies of acute liver failure, with acetaminophen toxicity being the most common cause (45.7% cases), followed by drug-induced idiosyncratic reactions, hepatitis B virus, ischemia, autoimmunity, hepatitis A virus, heat stroke, pregnancy-induced liver injury (acute fatty liver of pregnancy/HELLP syndrome), Bud-Chiari syndrome, Wilson’s disease, infiltrating malignancies [6].

Drug-induced liver injury is the second most common cause of acute liver failure after acetaminophen [7], with only 10% of patients developing acute liver failure with the potential of causing death [8]. The pathogenesis of this liver injury is believed to be immune-mediated, with certain individuals having a genetic predisposition, caused by specific HLA haplotypes [9]. Among various groups of drugs causing this liver injury, antibiotics account for 50% of these cases, partly because antibiotics usually require high doses but also because they are frequently used [10]. The patient described in the case also received cefepime and vancomycin for cellulitis, but his labs already reflected worsening liver function before he received the antibiotics. There are no specific diagnostic tests for drug-induced liver failure, with identification of the causative agent requiring thorough history collection and exclusion of other causes [11].

## Conclusions

Drug-induced liver injury remains the leading cause of acute hepatic failure in the United States. It is a diagnosis of exclusion and requires a high index of suspicion and detailed evaluation to attribute a drug as the underlying cause of liver damage. Daratumumab is well-documented to affect multiple organ systems, including bone marrow, gastrointestinal system, and nervous system, but has not been reported to cause fulminant hepatic failure, leading to encephalopathy and death. The awareness among oncologists and gastroenterologists of this serious adverse effect can help in early recognition and potentially preventing any serious harm to the patient.
